# Downregulated SPINK4 is associated with poor survival in colorectal cancer

**DOI:** 10.1186/s12885-019-6484-5

**Published:** 2019-12-30

**Authors:** Xiaojie Wang, Qian Yu, Waleed M. Ghareeb, Yiyi Zhang, Xingrong Lu, Ying Huang, Shenghui Huang, Yanwu Sun, Jiayi Lin, Jin Liu, Pan Chi

**Affiliations:** 1 Department of Colorectal Surgery, Union Hospital, Fujian Medical University, 29 Xin-Quan Road, Fuzhou, Fujian 350001 People’s Republic of China; 2 Department of Pathology, Union Hospital, Fujian Medical University, Fuzhou, People’s Republic of China; 3 Department of General and Gastrointestinal Surgery, Suez Canal University, Suez, Egypt; 4 Clinical Laboratory, Union Hospital, Fujian Medical University, Fuzhou, People’s Republic of China

**Keywords:** Colorectal cancer, SPINK4, Survival

## Abstract

**Background:**

SPINK4 is known as a gastrointestinal peptide in the gastrointestinal tract and is abundantly expressed in human goblet cells. The clinical significance of SPINK4 in colorectal cancer (CRC) is largely unknown.

**Methods:**

We retrieved the expression data of 1168 CRC patients from 3 Gene Expression Omnibus (GEO) datasets (GSE24551, GSE39582, GSE32323) and The Cancer Genome Atlas (TCGA) to compare the expression level of SPINK4 between CRC tissues and normal colorectal tissues and to evaluate its value in predicting the survival of CRC patients. At the protein level, these results were further confirmed by data mining in the Human Protein Atlas and by immunohistochemical staining of samples from 81 CRC cases in our own center.

**Results:**

SPINK4 expression was downregulated in CRC compared with that in normal tissues, and decreased SPINK4 expression at both the mRNA and protein levels was associated with poor prognosis in CRC patients from all 3 GEO datasets, the TCGA database and our cohort. Additionally, lower SPINK4 expression was significantly related to higher TNM stage. Moreover, in multivariate regression, SPINK4 was confirmed as an independent indicator of poor survival in CRC patients in all databases and in our own cohort.

**Conclusions:**

We concluded that reduced expression of SPINK4 relates to poor survival in CRC, functioning as a novel indicator.

## Highlight

- SPINK4 expression was downregulated in colorectal cancer compared with that in normal tissues.
- Decreased SPINK4 mRNA expression was associated with poor prognosis in colorectal cancer patients in 3 independent databases (GSE24551, GSE39582, the TCGA database) and our own cohort.
- This study is the first, to the best of our knowledge, to implicate SPINK4 as a novel indicator of poor survival in CRC.

## Background

Despite significant progress in surgery, radiotherapy, chemotherapy, and targeted therapy, colorectal cancer (CRC) remains one of the leading cancer types in terms of incidence and cancer-related death worldwide [1]. This characteristic is partly due to a lack of diagnostic markers for the detection of CRC and inefficient treatment of late-stage colorectal cancer [1]. Currently, the prediction of survival or relapse and the determination of therapeutic strategies are mostly based on the tumor-node-metastasis (TNM) system [2]. However, the long-term outcome varies widely, even in patients within the same TNM stage [3]. Moreover, this pathological prognostic prediction method alone may not accurately predict prognosis without incorporating molecular data of the tumor [4]. Hence, an increasing number of studies in this era of genomic medicine have focused on molecularly based prognostic markers, which are complementary to the pathological TNM system [5, 6].

Serine protease inhibitors function as central regulators of many vital processes in the mammalian body; when serine protease activity or serpin-mediated regulation becomes unbalanced or dysfunctional, severe disease states, such as cancer and sepsis, can ensue [7]. One branch of the family of serine protease inhibitors is named Kazal type (SPINK) and originally consisted of four members in humans (SPINK1, SPINK2, SPINK4, and SPINK5) [8]. Although the major site of expression of all four SPINK members may differ, all are thought to be involved in protection against proteolytic degradation of epithelial and mucosal tissues [8]. SPINK4 is abundantly expressed in human goblet cells but was also reported to be formed, stored, and secreted from monocytes and might function as a gastrointestinal peptide [9]. A previous study found that serum SPINK4 levels were increased in CRC and had high diagnostic value but were not associated with the survival of CRC patients [10]. The expression status of SPINK4 in tissue samples and its clinical significance in CRC is largely unknown. Therefore, the present study aimed to measure SPINK4 expression in CRC tissues and to investigate its relationship with clinicopathological features and survival.

## Methods

### Database analysis

A total of four microarray data sets were retrieved from the Gene Expression Omnibus (GEO) database (https://www.ncbi.nlm.nih.gov/geo/). The microarray data set GSE39582 included mRNA expression profiles of a large series of 443 CRC and 19 nontumor colorectal mucosa and was submitted by Nabila Elarouci et al. [11]. The microarray data set GSE24551 comprised two independent series including exon level expression profiling data for a total of 160 CRC tissue samples and was submitted by Anita Sveen et al. [12]. The microarray data set GSE32323 included mRNA expression profiles of 17 pairs of cancer and noncancerous tissues from colorectal cancer patients and was submitted by Kaoru Mogushi et al. [13]. The majority of colorectal cancers develop as tubular adenomas through multistage carcinogenesis. To address the sequential expression changes in normal colonic mucosa, adenoma, and carcinoma tissues, the mRNA expression profiles of 4 pairs of normal colonic mucosa and adenoma tissues and 4 pairs of adenoma and carcinoma tissues from GSE3880 were also downloaded.

The expression level of the SPINK4 gene in other cell lines, organs and cancers was identified in the MediSapiens IST Online database (http://ist.medisapiens.com/) and the online database Gene Expression Profiling Interactive Analysis (GEPIA) (http://gepia.cancer-pku.cn/index.html) [14].

RNA sequencing (RNA-Seq) data and the full clinicopathological dataset from 438 colon cancer patients from COAD were obtained from the TCGA data portal (https://portal.gdc.cancer.gov/). We excluded cases without sufficient survival data (*n* = 2), leaving 436 colon cancer patients selected for further survival analysis.

To further address the change in SPINK4 protein expression in CRC tissues, SPINK4 expression in CRC tissues and normal colon tissues was first reviewed by using the immunohistochemical (IHC) staining data provided in the Human Protein Atlas (http://www.proteinatlas.org/) [15].

Single-cell sequencing data and corresponding single-cell functional states from GSE81861 [16], which included the RNA expression profiles of 44 single CRC cells, were downloaded from CancerSEA [17].

**Fig. 1:**
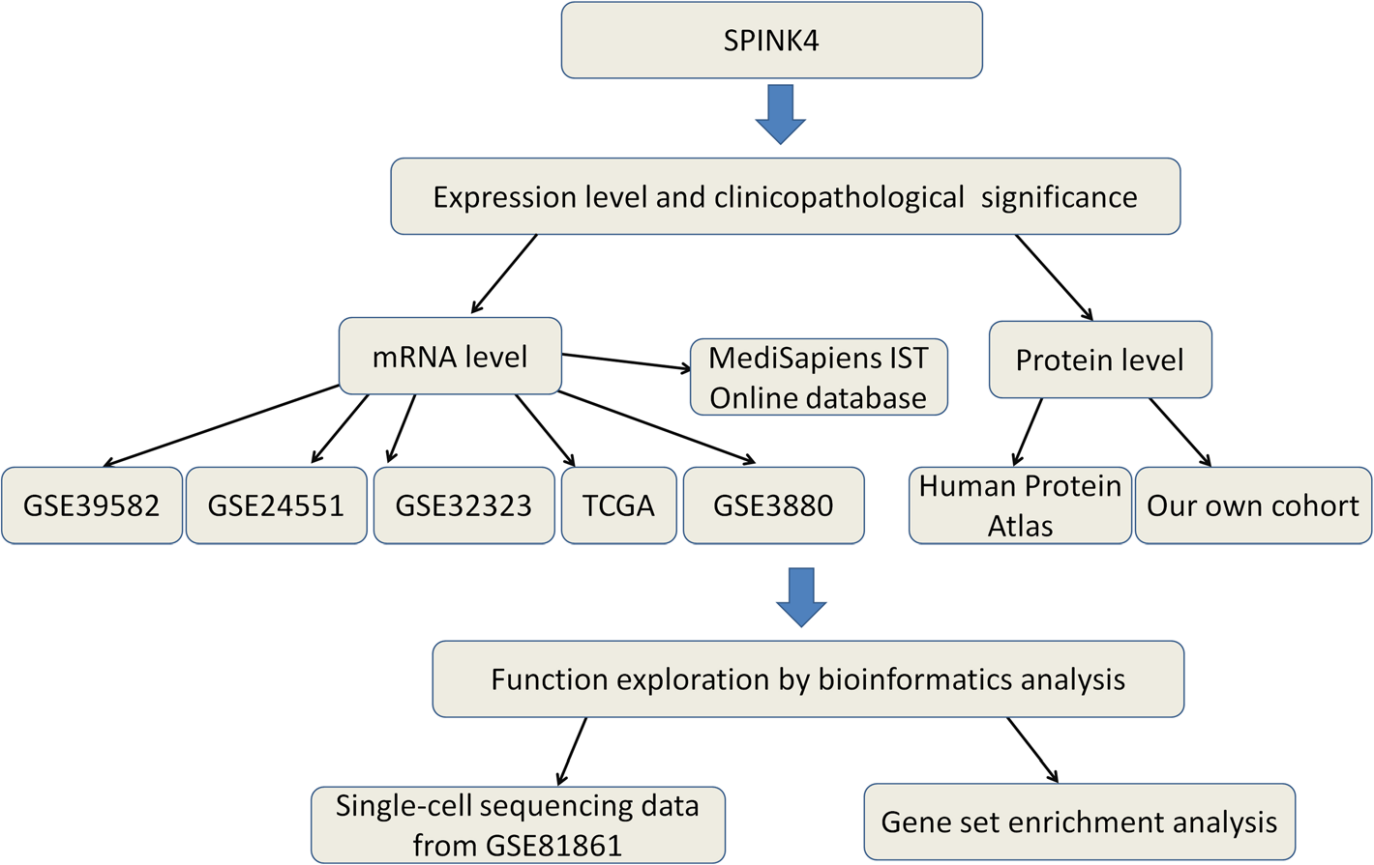
The workflow of this study

A workflow of this study is shown in Fig. 1.

### Tissues and relevant clinicopathological information of patients

A total of 81 paraffin-embedded colorectal cancer tissues and paired adjacent paraffin-embedded normal tissues were retrospectively collected to perform IHC staining. All patients had received radical surgery in the Department of Colorectal Surgery, Union Hospital, Fujian Medical University, between February 2012 and December 2013. The postsurgical pathologic diagnosis in all patients was adenocarcinoma. None of the patients received preoperative chemotherapy or radiotherapy. The exclusion criterion was the presence of other synchronous or metachronous tumors.

Tissue specimens were fixed in formalin and embedded in paraffin. All patients were followed up until May 2018. Detailed information on the clinical characteristics of all patients, including gender, age, body mass index (BMI), TNM stage, tumor size, histological type, pretreatment CEA level, pretreatment CA199 level, perineural invasion status, venous invasion status, tumor location, and tumor differentiation, was retrieved. Diagnosis and TNM staging were performed according to the 7th edition of the AJCC Cancer Staging Manual [18].

### IHC staining and interpretation of results

The differential protein expression levels of SPINK4 in 81 colorectal cancer and paired normal tissues were measured using IHC staining. Anti-SPINK4 monoclonal antibody (ab121257, Abcam, UK) was used at a working concentration of 1:200. The scores were evaluated based on staining intensity and the percentage of positive cells for each of the sections. The staining intensity was scored as follows: 0, no staining; 1, light yellow staining; 2, yellow-brown staining; and 3, deep brown staining. The percentage of positive cells was scored as follows: 0, 0~5%; 1, 6~25%; 2, 26~50%; 3, 51~75%; and 4, > 75%. The final score was calculated as follows: positive cell score × staining intensity score. The total scores were condensed into four categories: 0 for negative (−); 1–3 for weakly positive (+); 4–7 for positive (++); and 8–12 for strongly positive (+++). All patients were sorted into two groups according to the total score. High expression of SPINK4 was defined as a detectable immunoreaction with a total score of ≥1 + .

### Gene set enrichment analysis (GSEA)

To determine the function of SPINK4, GSEA was conducted in patients with the top 25% and with the bottom 25% of the expression in the GSE24551 dataset and GSE39582 dataset, respectively. The annotated gene set c2.cp.kegg.v5.2.symbols.gmt from the pathway database was selected as the reference gene set. *P* < 0.05, |enrichment score (ES) | > 0.3 and gene size ≥30 were set as the cutoff criteria. The overlapping enriched hallmark signatures in the GSE24551 dataset and GSE39582 dataset are illustrated by a Venn diagram.

### Statistical analysis

Categorical variables were compared using the χ2 test and Fisher’s exact test. Continuous variables were compared using Student’s t-test. The cutoff value for SPINK4 expression was assessed using X-tile 3.6.1 software (Yale University, New Haven, CT, USA) [19]. Survival curves were computed with the Kaplan-Meier method and compared using the log-rank test. Univariate Cox proportional hazards regression was applied to estimate the individual hazard ratios (HRs) for the survival rates. Significant variables in the univariate analysis (*P* < 0.05) were then retained in multivariate analysis using Cox proportional regression models to explore the independent indicators. To comprehensively explore which functional states are associated with SPINK4 at the single-cell level, a linear model was used to evaluate linear correlations between SPINK4 expression and 14 cancer-related functional states (stemness, invasion, metastasis, proliferation, EMT, angiogenesis, apoptosis, cell cycle, differentiation, DNA damage, DNA repair, hypoxia, inflammation and quiescence). A *P*-value of < 0.05 was set as the level of significance. All statistical analyses were performed using SPSS software (ver. 17, SPSS Inc., Chicago, IL, USA) and R (ver. 3.4.1).

## Results

### The mRNA expression of SPINK4 is downregulated in CRC tissues

**Fig. 2:**
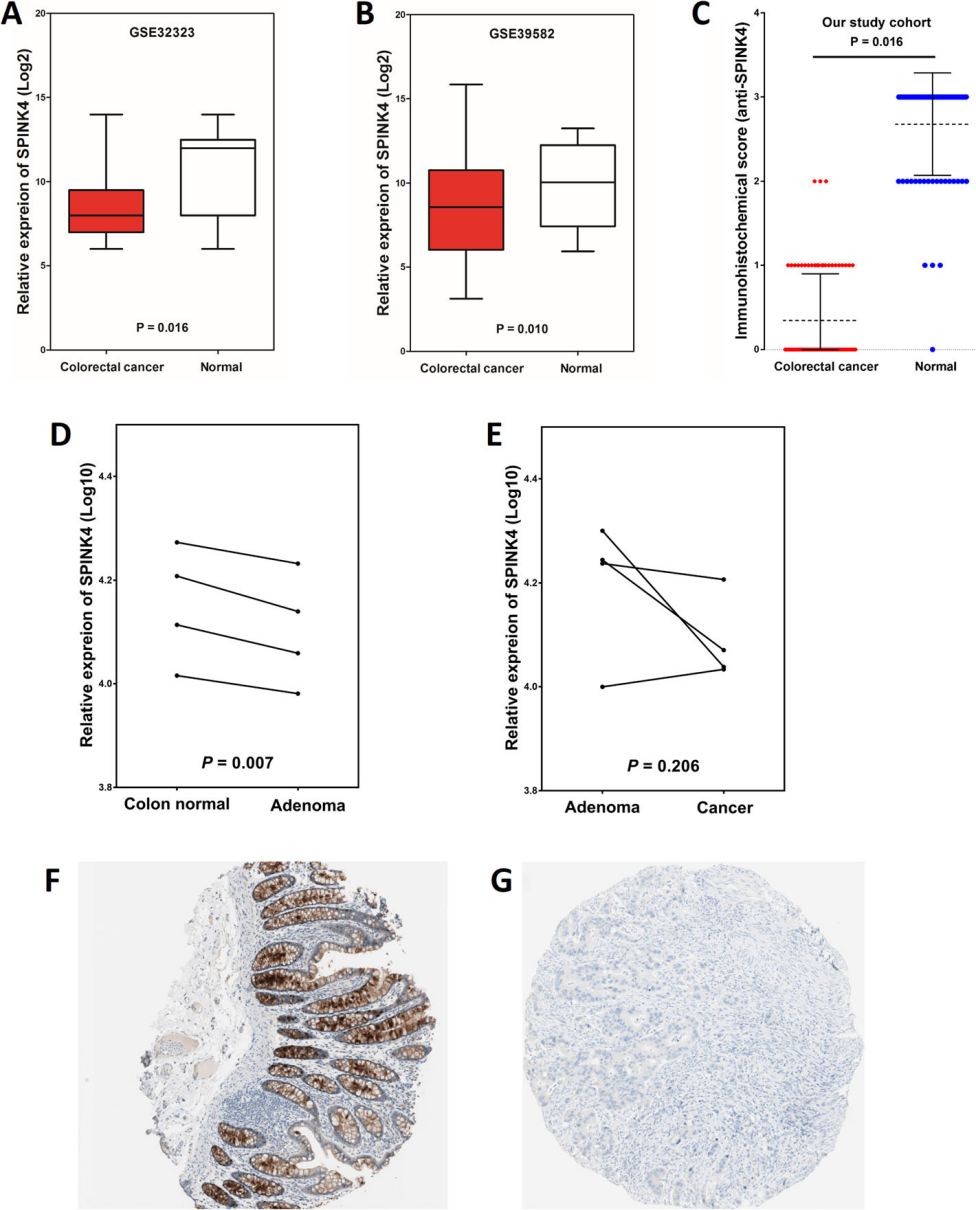
SPINK4 is downregulated in CRC. SPINK4 expression was significantly decreased in CRC tissues compared with that in paired adjacent normal tissues at the mRNA level in the (**a**) GSE32323 dataset and the (**b**) GSE39582 dataset and at the protein level in (**c**) our own cohort. **d** SPINK4 expression was decreased in adenoma tissues compared to that in adjacent normal mucosal tissues in GSE3880. **e** SPINK4 expression was similar between adenoma and carcinoma tissues in GSE3880. Validation of the expression of SPINK4 at the protein level in (**f**) normal and (**g**) CRC tissues by the Human Protein Atlas database (IHC staining)

To examine the levels of SPINK4 mRNA in CRC samples, we first analyzed SPINK4 mRNA expression by comparing 17 CRC tissues and paired adjacent normal tissues from the GSE32323 dataset, and the results indicated that the relative SPINK4 expression level was significantly decreased in CRC tissues compared with that in adjacent normal tissues (8.5 ± 2.2 vs. 10.5 ± 2.6, *P* = 0.016, Fig. 2a). These results were further confirmed in the GSE39582 dataset, in which the relative SPINK4 mRNA expression levels in CRC tissues and normal tissues were 8.5 ± 2.6 and 10.1 ± 2.3, respectively (*P* = 0.010, Fig. 2b). To address the sequential expression changes from normal colonic mucosa to adenoma to carcinoma, we analyzed SPINK4 mRNA expression by comparing 4 pairs of normal colonic mucosa and adenoma tissues and 4 pairs of adenoma and carcinoma tissues from the GSE3880 dataset. The results indicated that the relative SPINK4 expression level was decreased in adenoma compared to that in adjacent normal mucosa (4.1 ± 0.1 vs. 4.2 ± 0.1, *P* = 0.007, Fig. 2d). However, SPINK4 expression was similar between adenoma and carcinoma (4.1 ± 0.0 vs. 4.2 ± 0.1, *P* = 0.206, Fig. 2e).

### The mRNA expression of SPINK4 in other cell lines, organs and cancers

**Fig. 3:**
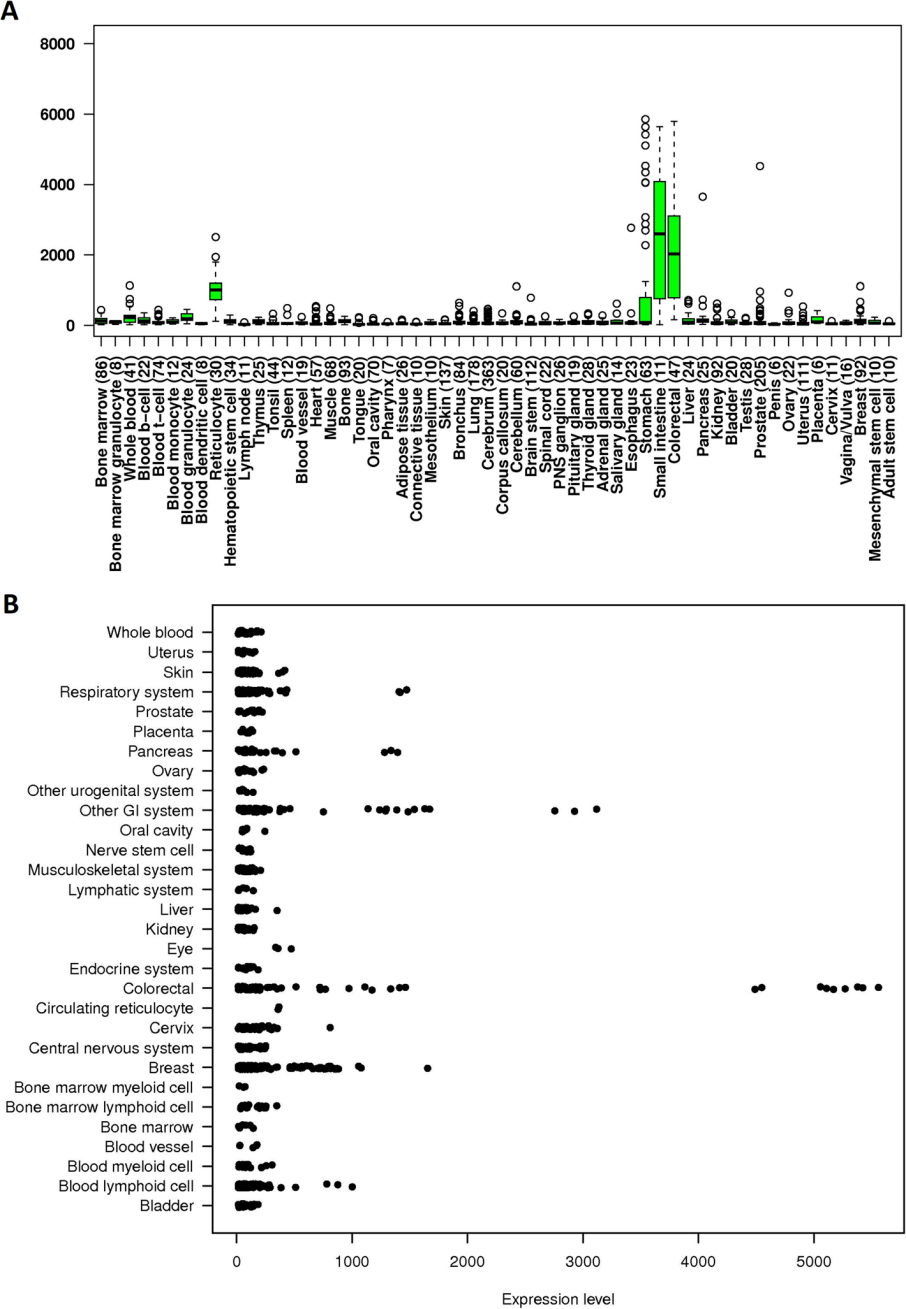
SPINK4 was highly expressed in normal colorectal, small intestinal and stomach tissues as well as in GI system cell lines, as evidenced by analysis of the IST Online database. **a** shows SPINK4 expression at tissue levels of different organs; **b** shows SPINK4 expression in different cell lines. GI system, gastrointestinal system

**Fig. 4:**
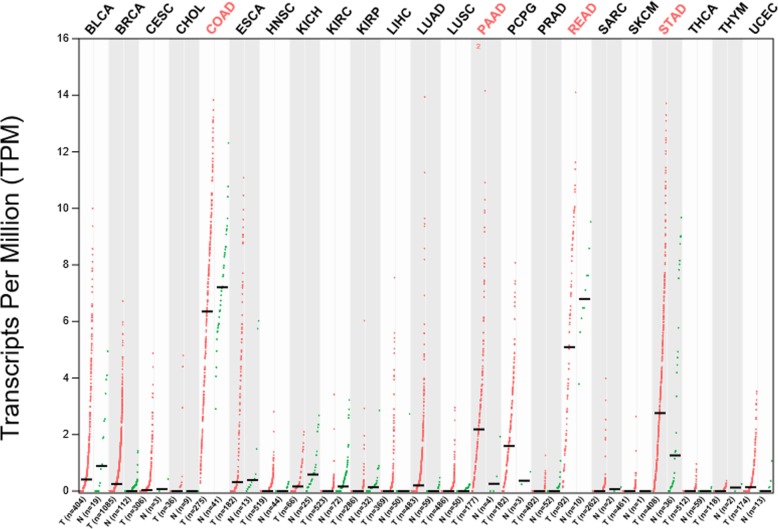
SPINK4 expression was higher in pancreatic adenocarcinoma (PAAD) and gastric adenocarcinoma (STAD) tissues than in the corresponding normal tissues, as evidenced by analysis of the GEPIA database. BLCA, Bladder Urothelial Carcinoma; BRCA, Breast invasive carcinoma; CESC, Cervical squamous cell carcinoma and endocervical adenocarcinoma; CHOL, Cholangio carcinoma; COAD, Colon adenocarcinoma; ESCA, Esophageal carcinoma; HNSC, Head and Neck squamous cell carcinoma; KICH, Kidney Chromophobe; KIRC, Kidney renal clear cell carcinoma; KIRP, Kidney renal papillary cell carcinoma; LIHC, Liver hepatocellular carcinoma; LUAD, Lung adenocarcinoma; LUSC, Lung squamous cell carcinoma; PAAD, Pancreatic adenocarcinoma; PCPG, Pheochromocytoma and Paraganglioma; PRAD, Prostate adenocarcinoma; READ, Rectum adenocarcinoma; SARC, Sarcoma; SKCM, Skin Cutaneous Melanoma; STAD, gastric adenocarcinoma; THCA, Thyroid carcinoma; THYM, Thymoma; UCEC, Uterine Corpus Endometrial Carcinoma

The SPINK4 mRNA levels in other various cell lines and normal organ tissues were analyzed via the IST Online database. This analysis revealed that SPINK4 was highly expressed in normal colorectal, small intestinal and stomach tissues as well as in gastrointestinal (GI) system cell lines (Fig. 3). In addition, the differences in SPINK4 expression in other tumor and normal tissues of multiple cancer types were analyzed in the GEPIA database. The results revealed that SPINK4 expression was higher in pancreatic adenocarcinoma (PAAD) and gastric adenocarcinoma (STAD) tissues than in the corresponding normal tissues (Fig. 4).

### The protein expression of SPINK4 was downregulated in CRC tissues

**Fig. 5:**
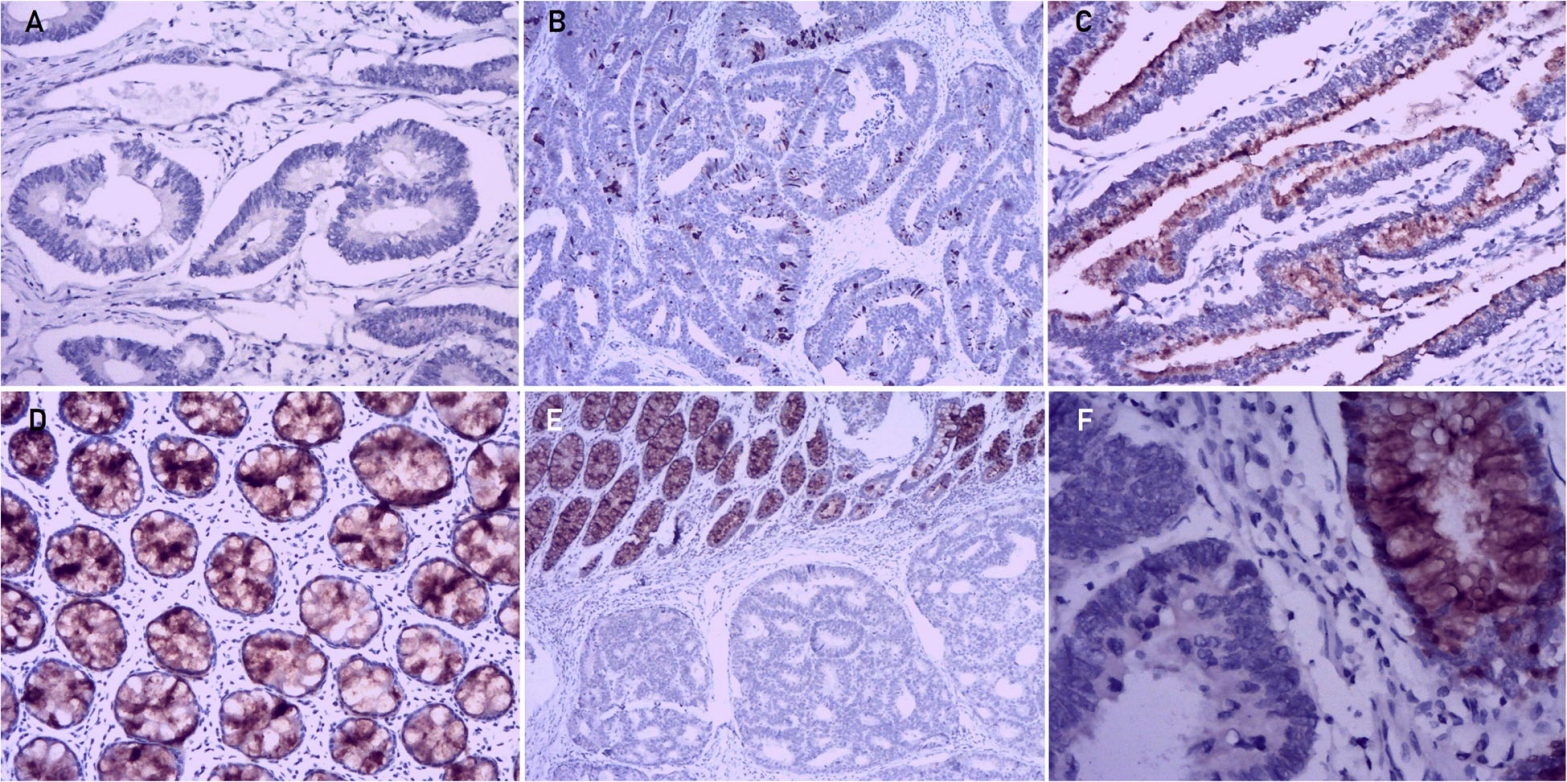
Representative images of SPINK4 immunohistochemical staining in CRC tissues from our study cohort. **a** Negative SPINK4 expression in CRC tissue (magnification × 200); **b** low SPINK4 expression in CRC tissue (magnification × 200); **c** moderate-strong SPINK4 expression in CRC tissue (magnification × 200); **d** strong SPINK4 expression in normal tissue. (magnification × 200); **e** Representative images of SPINK4 protein expression in sections of nonneoplastic mucosa adjacent to tumors (magnification × 200); **f** Representative images of SPINK4 protein expression in sections of nonneoplastic mucosa adjacent to tumors (magnification × 400)

To further address the change in SPINK4 protein expression in CRC tissues, data mining in the Human Protein Atlas was first performed. In all 3 normal colon tissues, SPINK4 expression was strongly positive, as determined by IHC staining, and was mainly located in the cytoplasm and membrane (Fig. 2f). However, among the 12 CRC tissues examined, 7 were negative for SPINK4 staining (negative SPINK4 staining rate: CRC tissues vs normal colon tissues: 58.3% vs 0%, Fig. 2g). Then, IHC staining was used to assess the levels of the SPINK4 protein in 81 colorectal cancer tissues compared with adjacent normal tissues. Positive expression of the SPINK4 protein was found in 98.8% (80/81) of the normal colorectal tissues and 30.9% (25/81) of the CRC tissues (*P* < 0.001). Moreover, the SPINK4 protein was expressed at significantly lower levels in CRC tissues (total score: 0.3 ± 0.5) than in normal tissues (total score: 2.7 ± 0.6; *P* = 0.016, Fig. 2c). SPINK4 was mainly located in the cytoplasm and membrane of normal mucosal epithelial cells and primary cancer cells. Images illustrating different SPINK4 expression levels in CRC tissues and paired adjacent normal tissues are shown in Fig. 5.

### Correlations between SPINK4 expression and clinicopathological characteristics

Subsequently, the correlations between SPINK4 expression and the clinicopathological characteristics of patients with CRC were investigated. Low SPINK4 expression was more frequently observed in patients with more advanced TNM stage (stage III-IV: 33/56, 58.9%) than in patients with lower TNM stage (stage I-II: 8/25, 32.0%, *P* = 0.025). In addition, low SPINK4 expression was significantly related to lower BMI (22.0 ± 3.4 vs. 23.9 ± 2.5, *P* = 0.031). SPINK4 expression was not associated with tumor grade, since the percentage of well to moderately differentiated tumors was similar between the low SPINK4 expression and high SPINK4 expression groups (96.4% vs. 92.0%, *P* = 0.583). No correlations were observed for gender, age, tumor size, histological type, pretreatment CEA level, pretreatment CA199 level, perineural invasion status, venous invasion status, or tumor location (Table 1).

**Table 1:** Correlations between SPINK4 expression and the clinicopathological features of patients with CRC in our own study cohort

	Low expression		High expression		*P* value
Gender					0.405
Male	28	(50.0)	15	(60.0)	
Female	28	(50.0)	10	(40.0)	
Age (years)	63.3 ± 12.4		64.1 ± 12.1		0.809
BMI (kg/m2)	22.0 ± 3.4		23.9 ± 2.5		0.031
TNM stage					0.025
I-II	23	(41.1)	17	(68.0)	
III-IV	33	(58.9)	8	(32.0)	
Tumor size (cm)	5.0 ± 2.0		4.1 ± 1.6		0.075
Histological type					0.366
Adenocarcinoma	53	(94.6)	22	(88.0)	
Mucinous adenocarcinoma	3	(5.4)	3	(12.0)	0.306
Pretreatment CEA level (ng/mL)	11.1 ± 22.1		11.4 ± 22.1		0.953
Pretreatment CA199 level (U/ml)	32.8 ± 75.3		17.5 ± 17.9		0.319
Perineural invasion	4	(7.1)	0	(0.0)	
Venus invasion	8	(14.3)	3	(12.0)	1.000
Tumor location					0.874
Left colon	5	(8.9)	3	(12.0)	
Right colon	19	(33.9)	9	(36.0)	
Rectum	32	(57.1)	13	(52.0)	
Tumor grade					0.583
Well to moderately differentiated	54	(96.4)	23	(92.0)	
Poorly differentiated	2	(3.6)	2	(8.0)	

### Correlations between SPINK4 expression and CRC patient survival

**Fig. 6:**
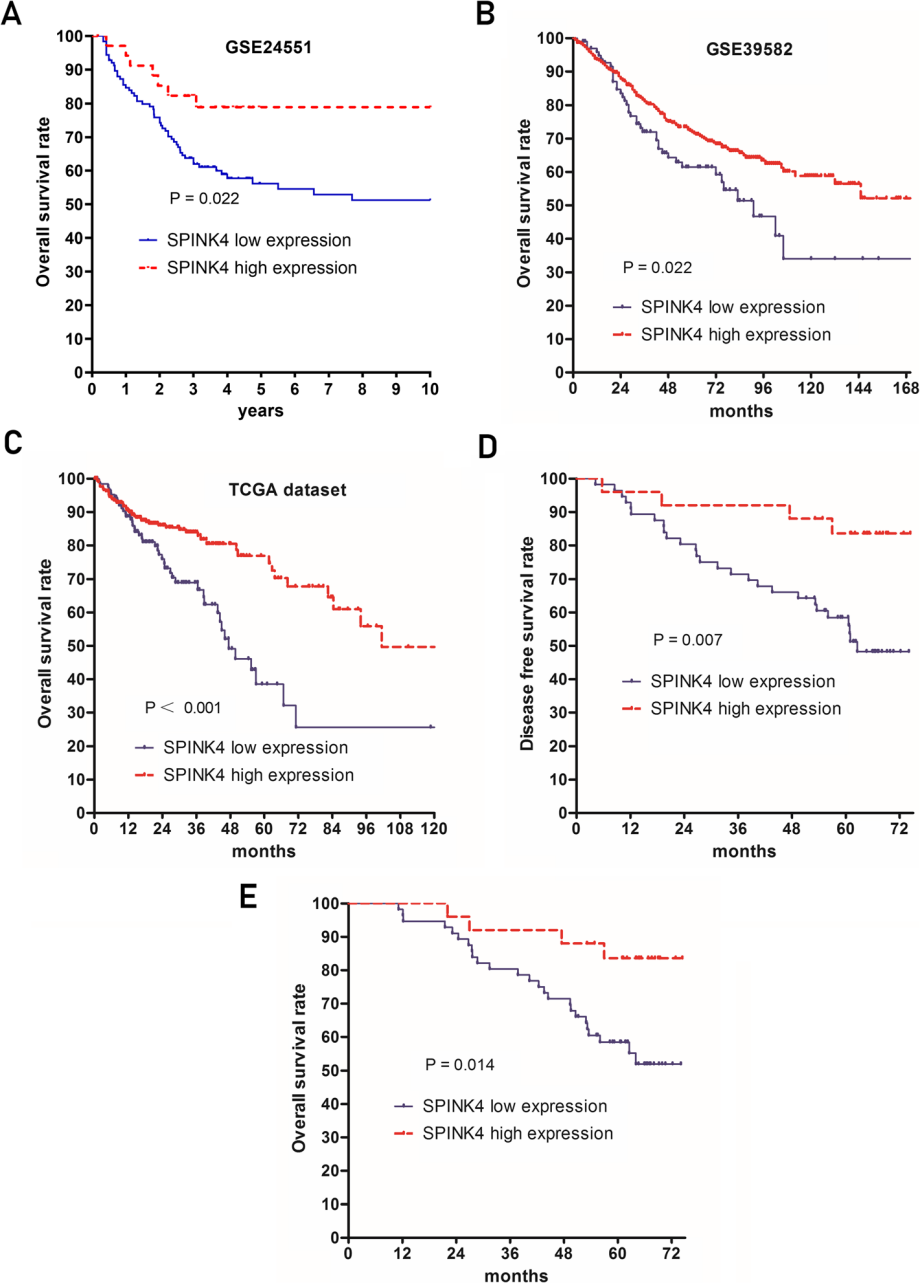
Low SPINK4 levels were associated with significantly decreased overall survival in CRC patients from **a** GSE24551, **b** GSE39582, **c** TCGA and **d** our study cohort. Low SPINK4 levels were associated with significantly decreased disease-free survival rates in CRC patients from **e** our study cohort

The prognostic significance of SPINK4 in CRC patients was explored first by a data mining approach in the GEO and TCGA databases at the mRNA level. The patient characteristics of each study cohort are summarized in Table 2. The study cohorts were divided into two groups according to the cutoff points, which were assessed using X-tile. CRC patients from the GSE24551 dataset with low SPINK4 mRNA levels had significantly lower 5-year overall survival rates (5Y-OS) than those with high SPINK4 mRNA levels (56.2% vs. 78.9%, *P* = 0.022, Fig. 6a). In the GSE39582 dataset, low SPINK4 mRNA expression was also significantly associated with decreased 5Y-OS in CRC patients (61.4% vs. 72.0%, *P* = 0.022, Fig. 6b). These results were also confirmed in patients from the TCGA database (5Y-OS of low expression vs. high expression: 38.5% vs. 76.9%, P<0.001, Fig. 6c). At the protein level, IHC staining was carried out in our own cohort, and survival analysis revealed that CRC patients with low levels of SPINK4 protein expression had significantly worse disease-free survival (DFS) and overall survival than those with high levels of SPINK4 protein expression (5Y-DFS of low expression vs. high expression: 58.5% vs. 83.6%, *P* = 0.007, Fig. 6d; 5Y-OS of low expression vs. high expression: 58.4% vs. 83.6%, *P* = 0.014, Fig. 6e). Furthermore, SPINK4 was confirmed as an independent indicator of poor survival in patients with CRC in multivariate Cox proportional hazard regression in all 3 databases (GSE24551, HR = 0.462, *P* = 0.056; GSE39582, HR = 0.636, *P* = 0.014; TCGA database, HR = 0.301, *P* = 0.014) and in our own cohort (HR = 0.299, *P* = 0.027 for OS; HR = 0.264, *P* = 0.014 for DFS) (Tables 3-4). In addition, other independent factors included TNM stage (GSE24551, GSE39582, TCGA, our cohort), age (GSE39582, TCGA, our cohort), microsatellite status (GSE24551), KRAS status (GSE39582), perineural invasion status (TCGA) and venous invasion status (TCGA).

**Table 2:** Clinical characteristics of patients in GSE24551, GSE39582 and TCGA database

Datasets	Characteristics		Values	
GSE24551 dataset	TNM stage	II	90	(56.3)
		III	70	(43.8)
	Microsatellite status	MSS, MSI-L	126	(78.8)
		MSI-H	21	(13.1)
		Missing	13	(8.1)
GSE39582 dataset	Gender	Male	288	(54.5)
		Female	240	(45.5)
	Age (years)		66.6 ± 13.4	
	TNM stage	0	4	(0.8)
		I	32	(6.1)
		II	246	(46.6)
		III	188	(35.6)
		IV	58	(11.0)
	BRAF status	WT	431	(81.6)
		MT	47	(8.9)
		Missing	50	(9.5)
	KRAS status	WT	305	(57.8)
		MT	202	(38.3)
		Missing	21	(4.0)
	TP53 status	WT	148	(28.0)
		MT	174	(33.0)
		Missing	206	(39.0)
	MMR status	pMMR	413	(78.2)
		dMMR	69	(13.1)
		Missing	46	(8.7)
TCGA database	Gender	Male	234	(53.4)
		Female	204	(46.6)
	Age (years)		66.6 ± 13.0	
	TNM stage	I	73	(16.7)
		II	194	(44.3)
		III	99	(22.6)
		IV	61	(13.9)
		Missing	11	(2.5)
	Pretreatment CEA level			
	(ng/ml)	38.4 ± 201.3		
	Histological type	Adenocarcinoma	373	(85.2)
		Mucinous adenocarcinoma	60	(13.7)
		Missing	5	(1.1)
	Perineural invasion	No	131	(29.9)
		Yes	45	(10.3)
		Missing	262	(59.8)
	Tumor deposits	No	184	(42.0)
		Yes	38	(8.7)
		Missing	216	(49.3)
	KRAS status	WT	24	(5.5)
		MT	22	(5.0)
		Missing	392	(89.5)

*MSS* Microsatellite Stable, *MSI-L* Microsatellite instability-low, *MSI-H* Microsatellite instability-high, *WT* Wide type, *MT* Mutation type, *DMMR* Mismatch repair deficient, *pMMR* Mismatch repair proficient

**Table 3:** Multivariate analysis of indicators for overall survival in CRC patients in GSE24551, GSE39582 and TCGA database

Datasets	Variable	Group	HR	(95% CI)		*P* value
GSE24551 dataset	Univariate analysis					
	TNM stage	(III vs. II)	1.966	1.190	3.246	0.008
	Microsatellite status	(MSI-H vs. MSS, MSI-L)	0.246	0.077	0.786	0.018
	SPINK4 expression	(high vs. low)	0.413	0.188	0.906	0.027
	Multivariate analysis					
	TNM stage	(III vs. II)	1.977	1.181	3.310	0.010
	Microsatellite status	(MSI-H vs. MSS, MSI-L)	0.231	0.072	0.738	0.013
	SPINK4 expression	(high vs. low)	0.462	0.210	1.018	0.056
GSE39582 dataset	Univariate analysis					
	Gender	(female vs. male)	0.773	0.569	1.051	0.100
	Age	(per year)	1.024	1.011	1.037	0.000
	TNM stage	(III-IV vs. I-II)	2.033	1.644	2.513	0.000
	MMR status	(dMMR vs. pMMR)	0.808	0.488	1.340	0.410
	TP53 status	(MT vs. WT)	1.290	0.884	1.883	0.187
	KRAS status	(MT vs. WT)	1.347	0.989	1.834	0.059
	BRAF status	(MT vs. WT)	1.162	0.681	1.982	0.582
	SPINK4 expression	(high vs. low)	0.662	0.464	0.944	0.023
	Multivariate analysis					
	Age	(per year)	1.025	1.012	1.039	0.000
	TNM stage	(III-IV vs. I-II)	1.921	1.406	2.625	0.000
	KRAS status	(MT vs. WT)	1.365	1.001	1.861	0.050
	SPINK4 expression	(high vs. low)	0.636	0.444	0.911	0.014
TCGA dataset	Univariate analysis					
	Gender	(female vs. male)	0.884	0.593	1.318	0.545
	Age	(per year)	1.018	1.002	1.035	0.030
	TNM stage	(III-IV vs. I-II)	3.002	1.979	4.554	0.000
	KRAS status	(MT vs. WT)	1.416	0.610	3.288	0.418
	Tumor deposits	(yes vs. No)	1.752	0.854	3.594	0.126
	Perineural invasion	(yes vs. No)	1.938	0.981	3.826	0.057
	Venus invasion	(yes vs. No)	2.566	1.658	3.970	0.000
	Pretreatment CEA level	(per ng/mL)	1.001	1.001	1.002	0.000
	Lymphatic invasion	(yes vs. No)	2.140	1.393	3.287	0.001
	Histological type	(mucinous adenocarcinoma vs. adenocarcinoma)	1.374	0.802	2.352	0.247
	SPINK4 expression	(high vs. low)	0.479	0.321	0.714	0.000
	Multivariate analysis					
	Age	(per year)	1.040	1.003	1.078	0.034
	TNM stage	(III-IV vs. I-II)	3.750	1.323	10.635	0.013
	Perineural invasion	(yes vs. No)	2.359	0.864	6.445	0.094
	Venous invasion	(yes vs. No)	2.773	1.066	7.217	0.037
	SPINK4 expression	(high vs. low)	0.301	0.116	0.785	0.014

MSS, microsatellite Stable; MSI-L, microsatellite instability-low; MSI-H, microsatellite instability-high; WT, wide type; MT, mutation type

**Table 4:** Multivariate analysis of indicators for survival in CRC patients in in our own cohort

Variable	Group	HR	(95% CI)		*P* value
Overall survival					
Univariate analysis					
Gender	(female vs. male)	0.583	0.275	1.236	0.159
Age	(per year)	1.037	1.004	1.070	0.026
BMI	(per kg/m2)	0.963	0.862	1.074	0.496
TNM stage	(per stage)	3.135	1.936	5.078	0.000
Tumor size	(per cm)	1.091	0.925	1.287	0.300
Pretreatment CEA level	(per ng/mL)	1.014	1.002	1.027	0.024
Pretreatment CA199 level	(per U/ml)	1.004	1.001	1.007	0.004
Histological type	(mucinous adenocarcinoma vs. adenocarcinoma)	1.994	0.693	5.739	0.201
Perineural invasion	(yes vs. No)	3.796	1.133	12.712	0.031
Venus invasion	(yes vs. No)	1.398	0.533	3.669	0.496
Tumor location		0.981	0.568	1.696	0.946
Differentiation	(well to moderately differentiated vs. poorly differentiated)	1.435	0.341	6.043	0.622
SPINK4 expression	(high vs. low)	0.289	0.100	0.832	0.021
Multivariate analysis					
Age	(per year)	1.034	1.003	1.066	0.031
TNM stage	(per stage)	3.093	1.873	5.109	0.000
SPINK4 expression	(high vs. low)	0.299	0.103	0.872	0.027
Disease-free survival					
Univariate analysis					
Gender	(female vs. male)	0.616	0.298	1.273	0.191
Age	(per year)	1.034	1.003	1.066	0.033
BMI	(per kg/m2)	0.962	0.867	1.069	0.474
TNM stage	(per stage)	3.104	1.943	4.957	0.000
Tumor size	(per cm)	1.099	0.936	1.291	0.247
Pretreatment CEA level	(per ng/mL)	1.012	1.000	1.024	0.059
Pretreatment CA199 level	(per U/ml)	1.004	1.001	1.007	0.003
Histological type	(mucinous adenocarcinoma vs. adenocarcinoma)	1.916	0.670	5.482	0.225
Perineural invasion	(yes vs. No)	2.918	0.873	9.757	0.082
Venus invasion	(yes vs. No)	1.534	0.629	3.741	0.347
Tumor location		1.051	0.617	1.792	0.855
Differentiation	(well to moderately differentiated vs. poorly differentiated)	1.424	0.340	5.975	0.629
SPINK4 expression	(high vs. low)	0.261	0.091	0.747	0.012
Multivariate analysis					
TNM stage	(per stage)	3.298	1.986	5.475	0.000
SPINK4 expression	(high vs. low)	0.264	0.091	0.766	0.014

### The potential functions of SPINK4 in CRC

To investigate the function of SPINK4 in CRC, single-cell sequencing data from GSE81861 were analyzed at the single-cell level. The relationships between SPINK4 expression and the 14 cellular functional states were evaluated by linear correlation analysis (Additional file 1: Figure S1A). The results showed that SPINK4 is significantly positively correlated with cell differentiation (r2 = 0.446, *P* = 0.002; Additional file 1: Figure S1B) and inflammation (r2 = 0.543, *P* < 0.001; Additional file 1: Figure S1C) but significantly negatively correlated with cell DNA repair (r2 = − 0.433, *P* = 0.003; Additional file 1: Figure S1D) and stemness (r2 = − 0.556, *P* < 0.001; Additional file 1: Figure S1E). Then, GSEA was conducted by analyzing data from the GSE24551 dataset and GSE39582 dataset. Nine hallmark gene sets, including “OXIDATIVE PHOSPHORYLATION”, “INOSITOL PHOSPHATE METABOLISM”, “ALZHEIMER’S DISEASE”, “MELANOGENESIS”, “PARKINSON’S DISEASE”, “FRUCTOSE AND MANNOSE METABOLISM”, “BUTANOATE METABOLISM”, “AMINO SUGAR AND NUCLEOTIDE SUGAR METABOLISM” and “PHOSPHATIDYLINOSITOL SIGNALING SYSTEM”, were enriched and shared by both GSE datasets and are suspected to be the crucial signatures of high SPINK4 expression (Additional file 3: Table S1, Additional file 2: Figure S2).

## Discussion

The family of SPINK protease inhibitors originally consisted of four members in humans: SPINK1, SPINK2, SPINK4, and SPINK5 [8]. SPINK1 is mainly produced in pancreatic acinar cells and is expressed in various cancers and inflammatory states. In addition to being a protease inhibitor, SPINK1 also acts as an acute-phase reactant and a growth factor. Furthermore, it has been shown to modulate apoptosis [20]. Ozaki et al. [21] suggested that SPINK1 stimulates the proliferation of pancreatic cancer cells through the EGFR/mitogen-activated protein kinase cascade. Ida et al. [22] demonstrated that SPINK1 stimulates the proliferation of colon cancer cells and is involved in colorectal cancer progression. Moreover, overexpression of SPINK1 is associated with adverse prognosis in other cancers, including prostate cancer [23], hepatocellular cancer [24] and breast cancer [25]. Thus, SPINK1 can be used as a prognostic tumor marker. However, there have been only a few studies on the gene encoding SPINK4, another member of the SPINK family, in tumors. The analysis in the present study revealed that SPINK4 was highly expressed in normal colorectal, small intestinal and stomach tissues as well as in GI system cell lines. We first investigated SPINK4 mRNA expression in tumors in data from the TCGA and two GEO datasets (GSE32323 and GSE39582), and the results showed that SPINK4 mRNA expression was significantly decreased in CRC tissues compared with that in paired adjacent normal tissues. In addition to being downregulated in CRC, SPINK4 expression was higher in pancreatic adenocarcinoma (PAAD) and gastric adenocarcinoma than in the corresponding normal tissues at the RNA level. The change in SPINK4 protein expression in CRC tissues was then validated by data mining of the Human Protein Atlas and by IHC staining in our own samples. Consistent with the predictive results in the database analysis, the SPINK4 protein was expressed at significantly lower levels in the 81 CRC tissues than in the paired normal tissues. Furthermore, SPINK4 mRNA expression was decreased in adenoma compared to that in adjacent normal mucosa in the present study. However, although the expression of SPINK4 in carcinoma tended to be further decreased compared to that in adenoma, the difference was not statistically significant. These results suggest that the decrease in SPINK4 expression is an early event in colon carcinogenesis. Due to the limited sample size of adenoma cases in the present study, whether SPINK4 can be used as a predictor of CRC formation requires further study. Interestingly, the serum SPINK4 level was increased in patients with CRC compared with that in healthy controls in previous research [10]. Since proteins are secreted by various cells as a response to various stimuli, determining whether serum SPINK4 is directly derived from the tumor needs further research. Previous cancer research demonstrated a very small overlap between malignant differentially expressed proteins in serum and tissue [26].

With regard to the value of serum SPINK4 for predicting survival, Xie et al. [10] did not find that serum SPINK4 was associated with OS or DFS in CRC patients. The short follow-up time (less than 10 months in half of the patients) as well as the small sample size in that study might partially explain the negative result. At the tissue level in the present study, IHC staining in our own cohort indicated that the expression level of the SPINK4 protein was significantly associated with reduced survival rates in patients with CRC. To obtain a reliable conclusion, these results were further externally validated in 3 other independent databases (TCGA and GSE24551, GSE39582). Additionally, in our multivariate Cox proportional hazard regression model, SPINK4 was confirmed as an independent indicator of poor survival in CRC patients in all 3 databases (GSE24551, GSE39582, TCGA) and in our own cohort. These findings suggest that SPINK4 may be exploited as a potential novel indicator of poor survival in patients with CRC.

The functional and pathway enrichment analysis of SPINK4 in CRC showed that biological processes such as oxidative phosphorylation, metabolism of some components, and process in Alzheimer’s disease were significantly enriched. In cancer cells, there is enhanced glucose use, with the rate of the tricarboxylic acid cycle and oxidative phosphorylation slowed and glycolysis increased, as a way to generate energy [27]. This metabolic switch provides substrates for cell growth and division and free energy. Blocking these metabolic pathways could lead to a new approach in cancer treatment [27]. The gene sets associated with oxidative phosphorylation-related pathways were enriched in the samples with high SPINK4 expression in the current study. Furthermore, at the single-cell level functional analysis in our study, SPINK4 was deregulated in cancer stem cells, which were demonstrated to show a distinct metabolic phenotype that can be highly glycolytic or oxidative phosphorylation-dependent [28]. Metabolic pathways, including inositol phosphate metabolism [29], fructose and mannose metabolism [30], and butanoate metabolism [31], were reported to be associated with cancer development. In the present study, the SPINK4 expression level was decreased in CRC. However, the process in Alzheimer’s disease pathway was significantly related to high expression of SPINK4. Both Alzheimer’s disease and cancer are prevalent in the elderly. Some epidemiological studies have reported a negative association between Alzheimer’s disease and cancer. The results of a meta-analysis suggested that individuals diagnosed with Alzheimer’s disease had a decreased risk for incident cancer by 42%, and patients with a history of cancer had a 37% decreased risk of Alzheimer’s disease [32]. However, the underlying mechanism is still unclear. Several basic studies have indicated that neurodegenerative disorders and cancer share several biological pathways that may contribute to this negative association [33]. For instance, deletion or mutation of Pin1 can induce Alzheimer’s disease-like pathological changes in mice [34]. However, Pin1 is overexpressed and/or activated by multiple mechanisms in many common human cancers and acts on multiple signaling pathways to promote tumorigenesis. Inhibition of Pin1 in animal models has profound antitumor effects [33]. Moreover, lower SPINK4 expression was found to be associated with CRC cell stemness properties and undifferentiated states in the present study. It was reported that HMGA1 promotes cancer stem cell properties and plays a role in the pathogenesis of Alzheimer’s disease [35]. In addition, higher DNA repair ability was related to lower SPINK4 levels in the single-cell analysis. This relationship could be explained by the observation that cancer stem cells have increased DNA repair abilities and demonstrate resistance to DNA-damaging treatment approaches [36]. These GSEA data and single-cell functional analyses provide directions for further research on the mechanism of SPINK4 in the development of CRC.

Our study has certain limitations. First, only small CRC tissue samples were collected retrospectively to investigate the impact of SPINK4 on the long-term survival of CRC patients, although the results were further externally validated in 3 other independent databases (TCGA, GSE24551, and GSE39582). More studies are needed to confirm the results. Second, the exact biological function of SPINK4 in CRC and its detailed molecular regulation mechanisms were not assessed in the present study. The hypothesis drawn from GSEA and the single-cell functional and sequencing data analyses needs to be further confirmed by in vitro and in vivo experiments.

## Conclusions

This preliminary study confirmed by using multiple datasets and our own database that reduced expression of SPINK4 relates to poor survival in CRC, functioning as a novel indicator.

## Supplementary information

**Additional file 1: Figure S1.** The functional states associated with SPINK4 at the single-cell level. (A) The heatmap displays correlations between SPINK4 and the functional states of cells: SPINK4 was significantly positively correlated with cell differentiation (B) and inflammation (C) and was significantly negatively correlated with cell DNA repair (D) and stemness (E).

**Additional file 2: Figure S2.** GSEA identified the most significant biological processes related to SPINK4 based on GSE39582 and GSE24551. GSE39582 and GSE24551 share 9 gene sets: “OXIDATIVE PHOSPHORYLATION”, “INOSITOL PHOSPHATE METABOLISM”, “ALZHEIMER’S DISEASE”, “MELANOGENESIS”, “PARKINSON’S DISEASE”, “FRUCTOSE AND MANNOSE METABOLISM”, “BUTANOATE METABOLISM”, “AMINO SUGAR AND NUCLEOTIDE SUGAR METABOLISM” and “PHOSPHATIDYLINOSITOL SIGNALING SYSTEM”.

**Additional file 3: Table S1.** The gene sets that were significantly associated with SPINK4 by Gene set enrichment analysis (GSEA)

## Data Availability

The dataset supporting the conclusions of this article is included within the article and its Additional file 3: Table S1. All TCGA related data can be obtained from the TCGA Data Portal via https://tcga-data.nci.nih.gov/. All GEO related data can be obtained from the GEO Data Portal via https://www.ncbi.nlm.nih.gov/geo/.

## Abbreviations

CRC: Colorectal cancer
GEO: Gene Expression Omnibus
GSEA: Gene Set Enrichment Analysis
IHC staining: Immunohistochemical staining
SPINK: Serine protease inhibitors, Kazal type
TCGA: The Cancer Genome Atlas
