# Supplementary figures and images for: Downregulated SPINK4 is associated with poor survival in colorectal cancer
Source: BMC Cancer. 2019 Dec 30;19:1258. doi: 10.1186/s12885-019-6484-5 (PMC6938003; doi:10.1186/s12885-019-6484-5)

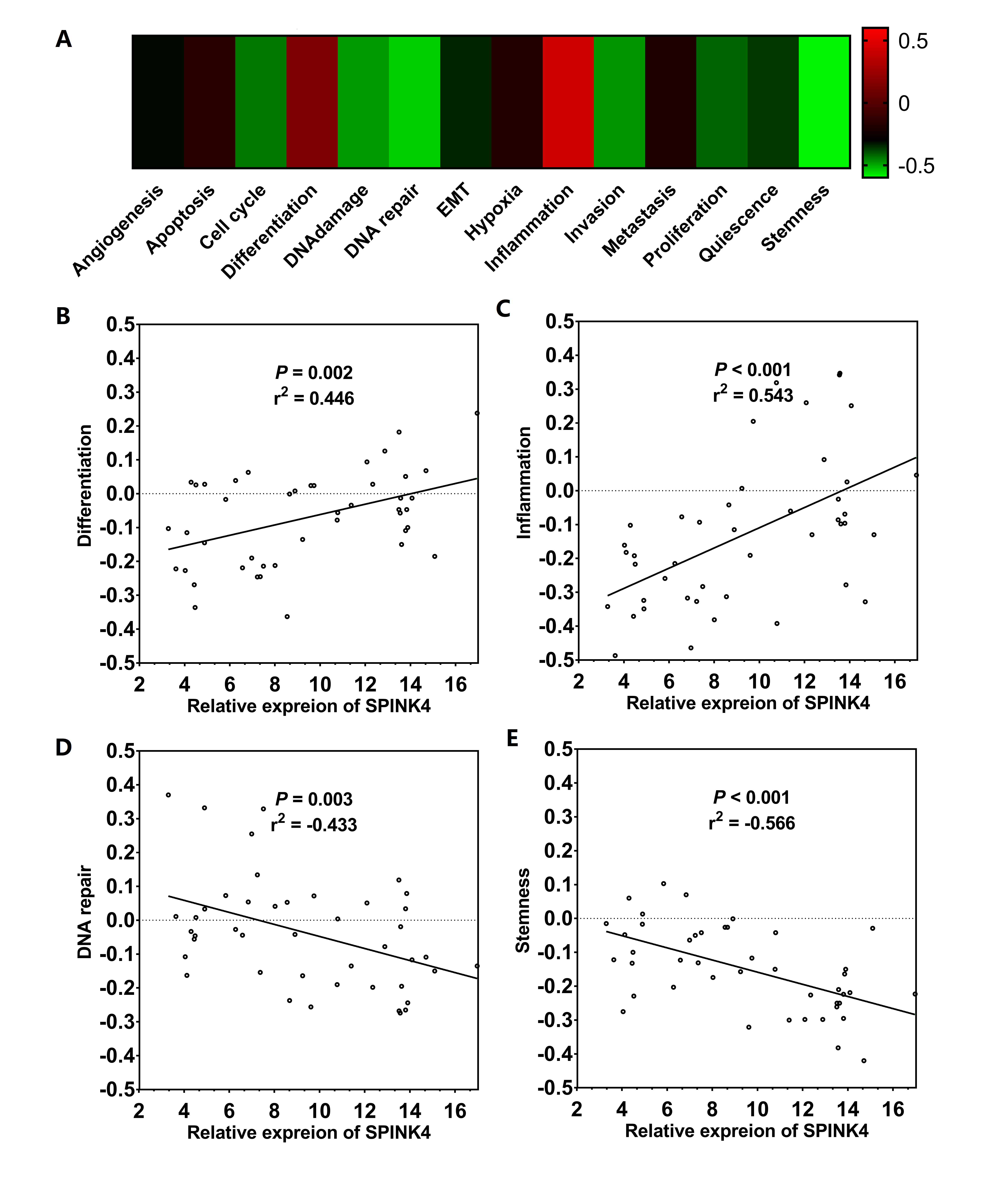

Supplement: Supplementary file 1 — Additional file 1: Figure S1. The functional states associated with SPINK4 at the single-cell level. (A) The heatmap displays correlations between SPINK4 and the functional states of cells: SPINK4 was significantly positively correlated with cell differentiation (B) and inflammation (C) and was significantly negatively correlated with cell DNA repair (D) and stemness (E). [file 12885_2019_6484_MOESM1_ESM.png]

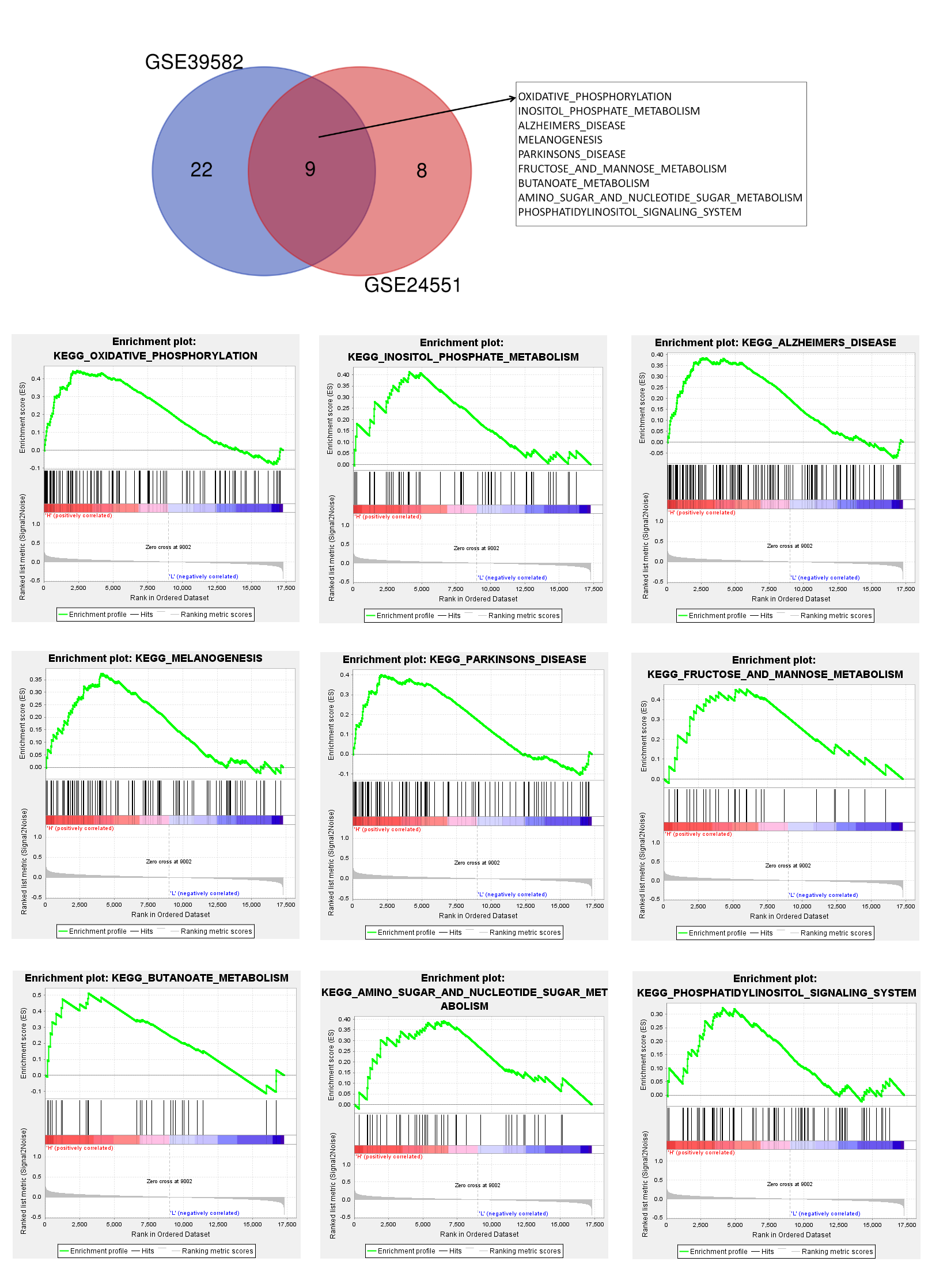

Supplement: Supplementary file 2 — Additional file 2: Figure S2. GSEA identified the most significant biological processes related to SPINK4 based on GSE39582 and GSE24551. GSE39582 and GSE24551 share 9 gene sets: “OXIDATIVE PHOSPHORYLATION”, “INOSITOL PHOSPHATE METABOLISM”, “ALZHEIMER’S DISEASE”, “MELANOGENESIS”, “PARKINSON’S DISEASE”, “FRUCTOSE AND MANNOSE METABOLISM”, “BUTANOATE METABOLISM”, “AMINO SUGAR AND NUCLEOTIDE SUGAR METABOLISM” and “PHOSPHATIDYLINOSITOL SIGNALING SYSTEM”. [file 12885_2019_6484_MOESM2_ESM.png]
