# Supplementary material for: Downregulated SPINK4 is associated with poor survival in colorectal cancer
Source: BMC Cancer. 2019 Dec 30;19:1258. doi: 10.1186/s12885-019-6484-5 (PMC6938003; doi:10.1186/s12885-019-6484-5)
Supplement: Supplementary file 3 — Additional file 3: Table S1. The gene sets that were significantly associated with SPINK4 by Gene set enrichment analysis (GSEA) [file 12885_2019_6484_MOESM3_ESM.docx]

Supplementary Table 1 The gene sets that were significantly associated with SPINK4 by Gene set enrichment analysis (GSEA)

|  | GSE24551 |  |  | GSE39582 |  |  |
| --- | --- | --- | --- | --- | --- | --- |
|  | Gene size | Enrichment score | P value | Gene size | Enrichment score | P value |
| OXIDATIVE_PHOSPHORYLATION | 106 | 0.445 | 0.004 | 116 | 0.424 | 0.030 |
| INOSITOL_PHOSPHATE_METABOLISM | 54 | 0.413 | 0.006 | 54 | 0.451 | 0.023 |
| ALZHEIMERS_DISEASE | 148 | 0.386 | 0.006 | 152 | 0.456 | 0.004 |
| MELANOGENESIS | 101 | 0.375 | 0.010 | 96 | 0.411 | 0.048 |
| PARKINSONS_DISEASE | 103 | 0.401 | 0.015 | 112 | 0.530 | 0.006 |
| FRUCTOSE_AND_MANNOSE_METABOLISM | 32 | 0.453 | 0.020 | 33 | 0.641 | 0.002 |
| BUTANOATE_METABOLISM | 30 | 0.512 | 0.027 | 33 | 0.661 | 0.018 |
| AMINO_SUGAR_AND_NUCLEOTIDE_SUGAR_METABOLISM | 42 | 0.392 | 0.028 | 42 | 0.513 | 0.027 |
| PHOSPHATIDYLINOSITOL_SIGNALING_SYSTEM | 76 | 0.325 | 0.037 | 74 | 0.401 | 0.038 |
